# Supplementary material for: Neural cell integration into 3D bioprinted skeletal muscle constructs accelerates restoration of muscle function
Source: Nat Commun. 2020 Feb 24;11:1025. doi: 10.1038/s41467-020-14930-9 (PMC7039897; doi:10.1038/s41467-020-14930-9)
Supplement: Supplementary file 1 — Supplementary Information [file 41467_2020_14930_MOESM1_ESM.pdf]

# Supplementary Information

## Neural Cell Integration into 3D Bioprinted Skeletal Muscle Constructs Accelerates Restoration of Muscle Function

Kim et al.

**Supplementary Table 1.** Quantification of human growth factors and cytokines detected in the hMPC- and hNSC-conditioned media.

| **Growth factors/cytokines** | **MPCs (pg per ml)** | **NSCs (pg per ml)** |
|---|---|---|
| BMP-4 | 0.0* | 7.4 |
| EGF | 0.0* | 6.4 |
| EGF R | 250.7 | 273.0 |
| FGF-2 | 0.0* | 3.4 |
| FGF-4 | 0.0* | 57.3 |
| IGFBP-2 | 7744.7 | 25453.9† |
| Insulin | 0.0* | 2924.2 |
| OPN | 1483.8 | 3232.5 |
| PDGF-AB | 4.9 | 124.2 |
| PDGF-BB | 3.9 | 70.1 |
| PDGF Rb | 81.6 | 301.7 |
| VCAM-1 | 829.5 | 2462.8 |
| VEGF R2 | 2.9* | 11.8 |

BMP-4: bone morphogenetic protein 4, EGF: epidermal growth factor, EGF R: epidermal growth factor receptor, FGF: fibroblast growth factor, IGFBP-2: insulin-like growth factor-binding protein 2, OPN: osteopontin, PDGF: platelet-derived growth factor, PDGF Rb: platelet-derived growth factor receptor-beta, VCAM-1: vascular cell adhesion protein 1, VEGF R2: vascular endothelial growth factor receptor 2. *Values below the limit of detection, and †values above the highest standards.

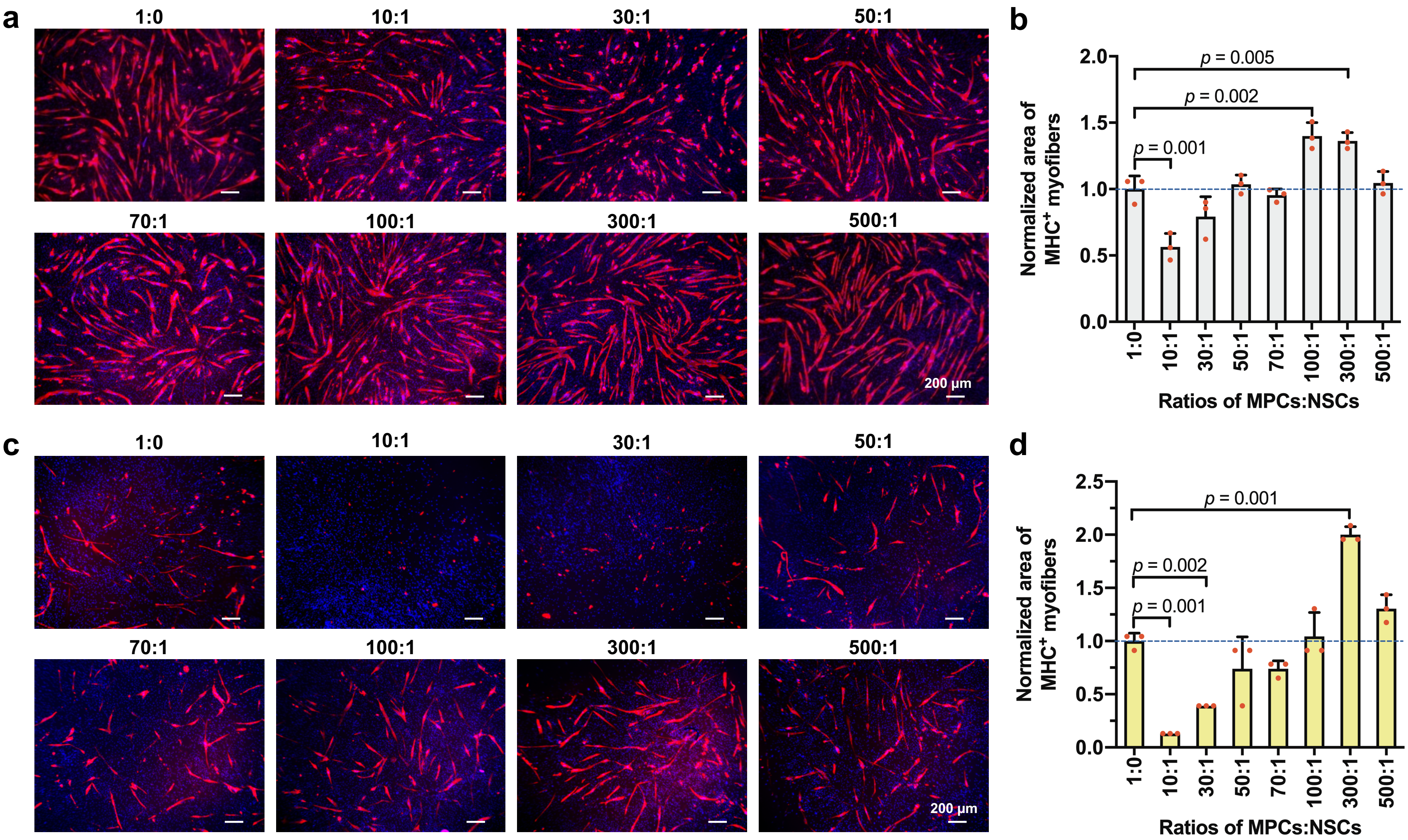


**Supplementary Fig. 1**. Myotube formation in 2D co-culture of hMPCs and hNSCs. Myotube formation and long-term survival were evaluated in different hMPCs:hNSCs ratios from 1:0 to 500:1 at 5 and 10 days of differentiation. Immunofluorescence for MHC

(red)/DAPI (blue) was performed at **a** 5 days and **c** 10 days in culture. The experimental findings were qualitatively reproduced three times. Quantitative data of the area of $MHC^+$ myofibers (normalized) at **b** 5 days and **d** 10 days in cuture ($n = 3$ per group). All data are represented as mean ± SD. The *p*-values by one-way ANOVA followed by Tukey's test are indicated.

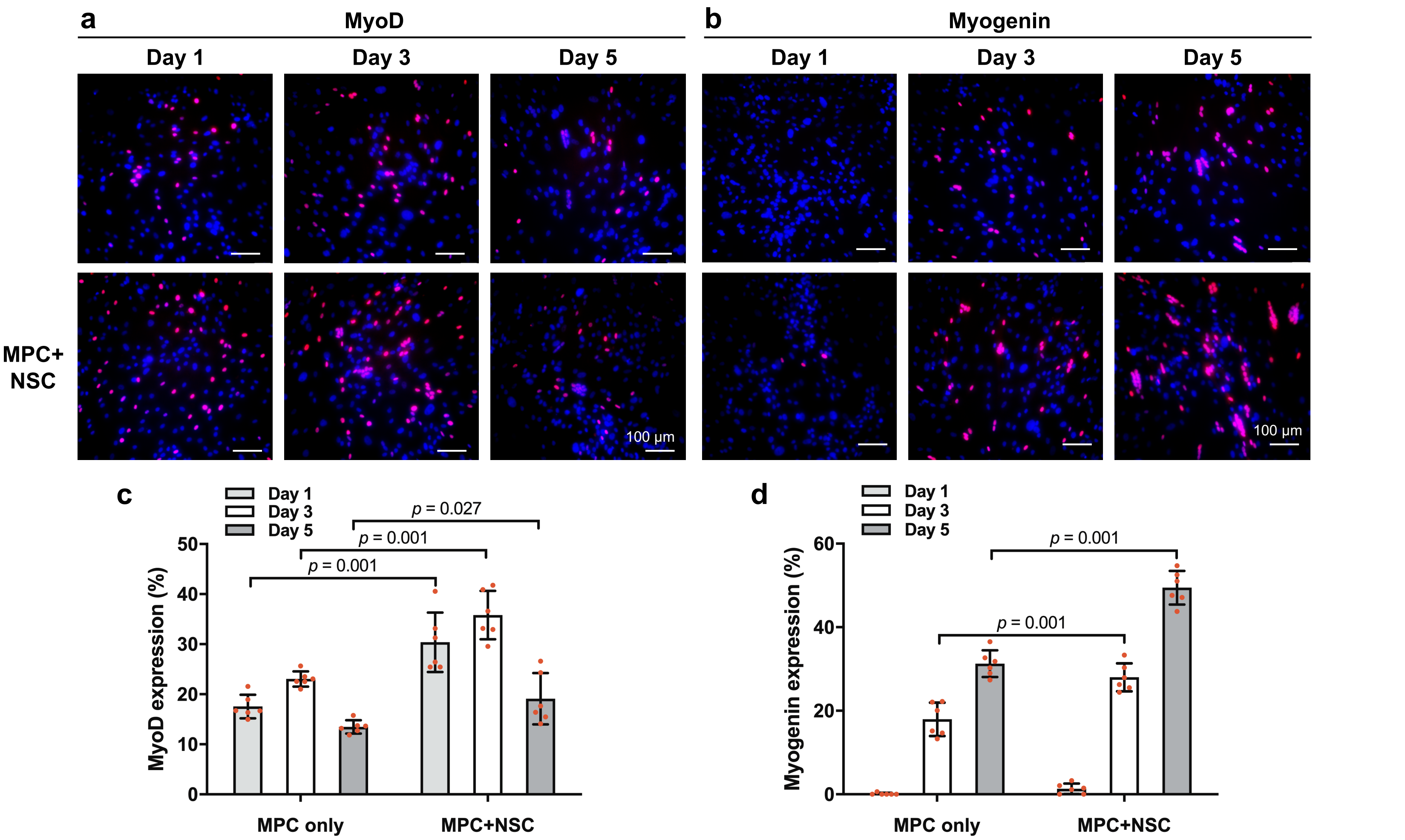


**Supplementary Fig. 2.** Immunofluorescence for **a** myoD and **b** myogenin at 1, 3, and 5 days in culture. The experimental findings were qualitatively reproduced three times. Quantification of **c** myoD and **d** myogenin expression in MPC and MPC+NSC (300:1) ($n$ = 6 per group and time point). All data are represented as mean ± SD. The $p$-values by two-way ANOVA followed by Tukey's test are indicated.

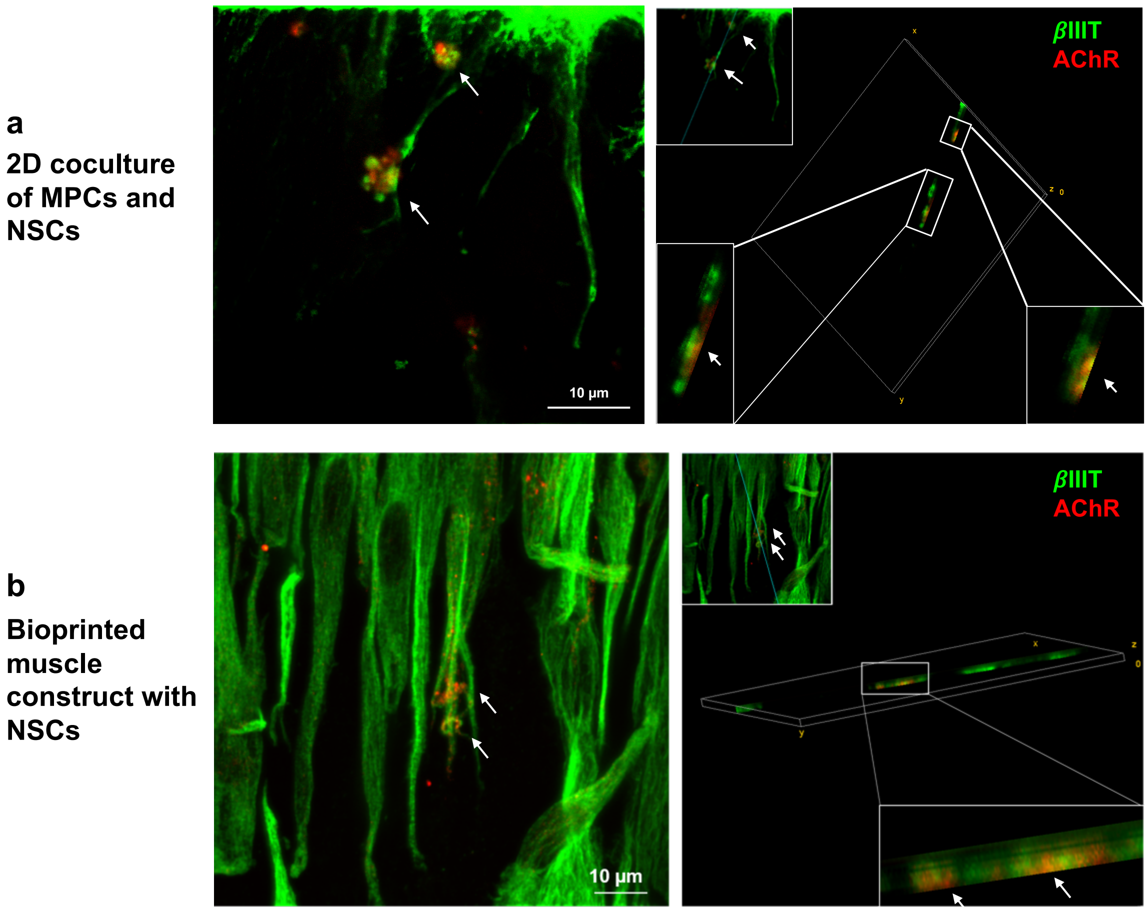


**Supplementary Fig. 3.** Neuromuscular junction formation. Immunofluorescence for βIIIT (green)/AChR (red) on (A) 2D co-culture and (B) bioprinted skeletal muscle construct (MPC+NSC). *Z*-stack confocal microscopy image (arrows, $\beta IIIT^{+}$ $AChR^{+}$ neuromuscular junctions). The experimental findings were qualitatively reproduced three times.

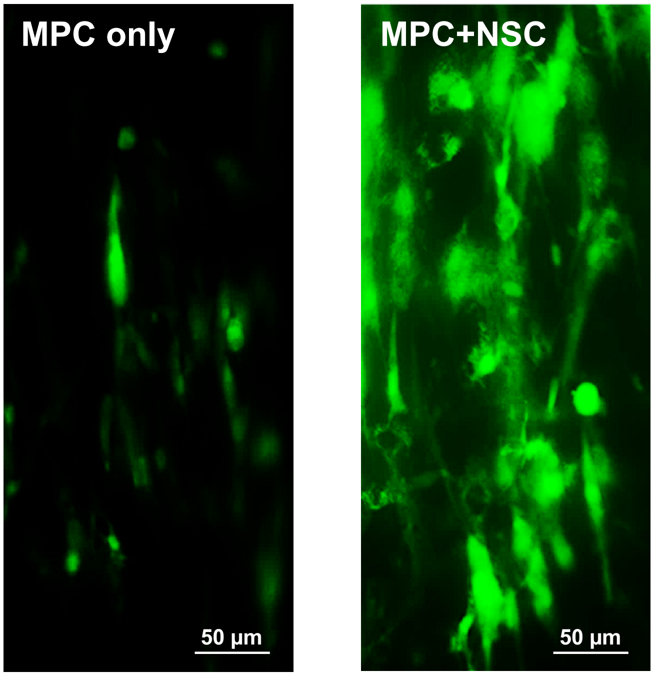


**Supplementary Fig. 4.** Calcium uptake images of the bioprinted skeletal muscle constructs (MPC only vs. MPC + NSC). Green fluorescence indicates intracellular calcium ions. The experimental findings were qualitatively reproduced three times.

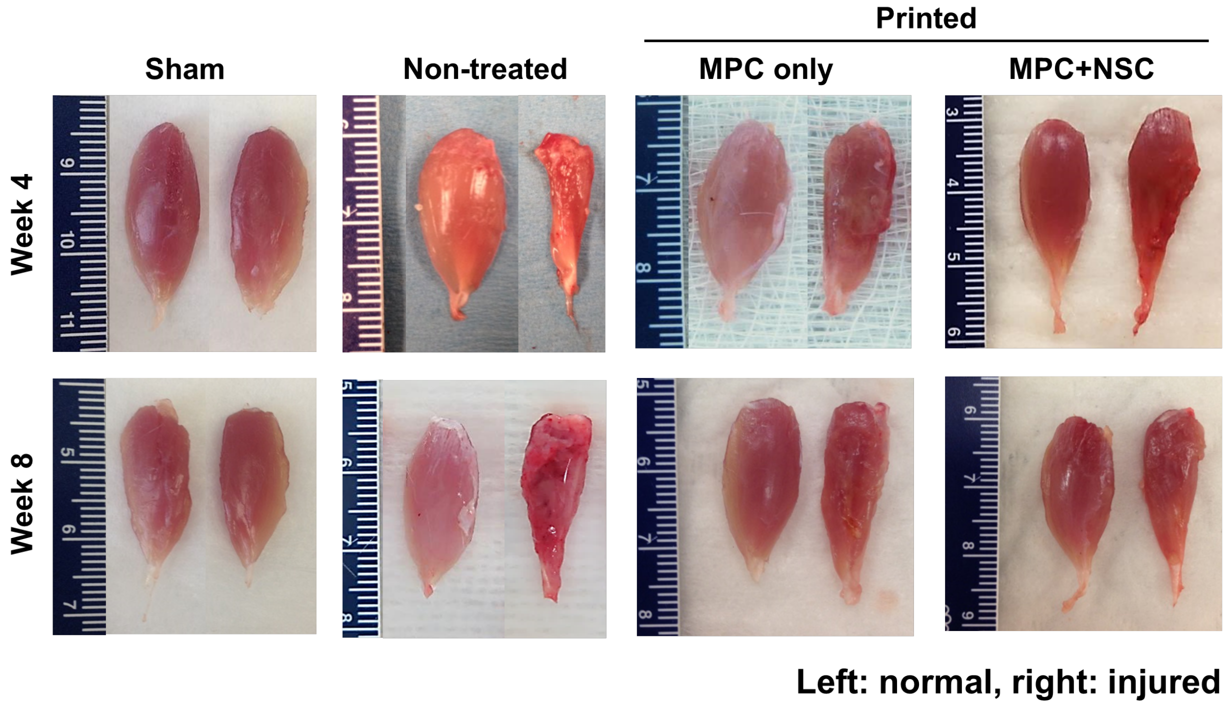


**Supplementary Fig. 5**. Gross appearance of TA muscles in the rat model of TA defect injury. The muscle defect was created by excising 40% of the TA muscle of the left leg; bioprinted skeletal muscle constructs were implanted into the defect sites. Harvested TA muscles of left legs (defective TA, right) and right legs (contralateral normal TA, left) at 4 and 8 weeks after implantation. The experimental findings were qualitatively reproduced three times.
