# Supplementary material for: Neural cell integration into 3D bioprinted skeletal muscle constructs accelerates restoration of muscle function
Source: Nat Commun. 2020 Feb 24;11:1025. doi: 10.1038/s41467-020-14930-9 (PMC7039897; doi:10.1038/s41467-020-14930-9)
Supplement: Supplementary file 3 — Reporting Summary [file 41467_2020_14930_MOESM3_ESM.pdf]

nature research

Corresponding author(s): Sang Jin Lee

Last updated by author(s): Jan 25, 2020

Reporting Summary

Nature Research wishes to improve the reproducibility of the work that we publish. This form provides structure for consistency and transparency in reporting. For further information on Nature Research policies, see Authors & Referees and the Editorial Policy Checklist.

Statistics

For all statistical analyses, confirm that the following items are present in the figure legend, table legend, main text, or Methods section.

n/a	Confirmed	
☐	☒	The exact sample size (*n*) for each experimental group/condition, given as a discrete number and unit of measurement
☐	☒	A statement on whether measurements were taken from distinct samples or whether the same sample was measured repeatedly
☐	☒	The statistical test(s) used AND whether they are one- or two-sided<br>*Only common tests should be described solely by name; describe more complex techniques in the Methods section.*
☐	☒	A description of all covariates tested
☐	☒	A description of any assumptions or corrections, such as tests of normality and adjustment for multiple comparisons
☐	☒	A full description of the statistical parameters including central tendency (e.g. means) or other basic estimates (e.g. regression coefficient) AND variation (e.g. standard deviation) or associated estimates of uncertainty (e.g. confidence intervals)
☐	☒	For null hypothesis testing, the test statistic (e.g. *F*, *t*, *r*) with confidence intervals, effect sizes, degrees of freedom and *P* value noted<br>*Give P values as exact values whenever suitable.*
☒	☐	For Bayesian analysis, information on the choice of priors and Markov chain Monte Carlo settings
☒	☐	For hierarchical and complex designs, identification of the appropriate level for tests and full reporting of outcomes
☒	☐	Estimates of effect sizes (e.g. Cohen's *d*, Pearson's *r*), indicating how they were calculated

Our web collection on statistics for biologists contains articles on many of the points above.

Software and code

Policy information about availability of computer code

Data collection	FV10-ASW 04.02., Image Pro 6.3., LAS-AF 3.1.8976.3, cellSens Dimension 1.18, MI-RAT 2.74
Data analysis	Image J 1.8.0_112 (https://imagej.nih.gov/ij/download.html), SPSS software 19, Origin pro 8.5, GraphPad Prism 5

For manuscripts utilizing custom algorithms or software that are central to the research but not yet described in published literature, software must be made available to editors/reviewers. We strongly encourage code deposition in a community repository (e.g. GitHub). See the Nature Research guidelines for submitting code & software for further information.

Data

Policy information about availability of data

All manuscripts must include a data availability statement. This statement should provide the following information, where applicable:

- Accession codes, unique identifiers, or web links for publicly available datasets
- A list of figures that have associated raw data
- A description of any restrictions on data availability

The data had been included in the manuscript or its supplementary material. The additional data generated or analysed during this study are available from the corresponding author on request.

Field-specific reporting

Please select the one below that is the best fit for your research. If you are not sure, read the appropriate sections before making your selection.

☒ Life sciences ☐ Behavioural & social sciences ☐ Ecological, evolutionary & environmental sciences

Life sciences study design

All studies must disclose on these points even when the disclosure is negative.

Sample size	No sample size calculation was performed in this study. Sample size was determined to be adequate based on the magnitude and consistency of measurable differences between groups. In our previous study (Kim, J.H. et al. 3D Bioprinted Human Skeletal Muscle Constructs for Muscle Function Restoration. Scientific reports 8, 12307 (2018) - https://www.nature.com/articles/s41598-018-29968-5) and this study, the statistically significant differences among groups had showed with the determined sample size. Detail of sample size of all experiments were provided in the text and figure legends.
Data exclusions	On principle, data were only excluded for failed experiments, reasons for microbial contamination of in vitro experiments.
Replication	The experimental findings (Fig. 3h-3i, Supplementary Fig. S2, S4 and S5) were qualitatively reproduced three times. Replicate experiments were successful.
Randomization	For the in vivo study, recipient rats were allocated randomly for the engineered tissue implantation of Printed-MPC, Printed-MPC+NSC, or non-treated (defect only without implantation).
Blinding	The investigators were blinded to group allocation during data collection and analysis.

Reporting for specific materials, systems and methods

We require information from authors about some types of materials, experimental systems and methods used in many studies. Here, indicate whether each material, system or method listed is relevant to your study. If you are not sure if a list item applies to your research, read the appropriate section before selecting a response.

Materials & experimental systems

n/a	Involved in the study	
☐	☒	Antibodies
☐	☒	Eukaryotic cell lines
☒	☐	Palaeontology
☐	☒	Animals and other organisms
☒	☐	Human research participants
☒	☐	Clinical data

Methods

n/a	Involved in the study	
☒	☐	ChIP-seq
☒	☐	Flow cytometry
☒	☐	MRI-based neuroimaging

Antibodies

Antibodies used	mouse anti-MF-20 antibody (MF-20, 1 µg/ml, Developmental Studies Hybridoma Bank), mouse anti-myoD (MA1-41017, 1:200 dilution, Thermo Scientific Inc.), rabbit anti-myogenin (ab124800, 1:200 dilution, Abcam), rabbit anti-βIIIT (ab18207, 1:100 dilution, Abcam), rabbit anti-NF (N4142, 1:80 dilution, MilliporeSigma), rabbit anti-GFAP (ab7260, 1:100 dilution, Abcam), rat anti-AChR antibody (ab24719, 1:100 dilution, Abcam), rabbit anti-vWF (A0082, 1:400 dilution, Dako) and mouse anti-α-SMA (sc-53015, 1:50 dilution, Santa Cruz Biotechnology), chicken anti-NF (ab4680, 1:1000 dilution, Abcam), rabbit anti-HLA (ab52922, 1:100 dilution, EMD Millipore), mouse anti-HNA (clone 3E1.3, MAB4383, 1:100 dilution, EMD Millipore), Texas Red-conjugated anti-mouse IgG (TI-2000, 1:200 dilution, Vector Labs), Texas Red-conjugated anti-rabbit IgG (TI-1000, 1:200 dilution, Vector Labs) Texas Red-conjugated anti-rat IgG (TI-9400, 1:200 dilution, Vector Labs), Alexa 488-conjugated anti-rabbit IgG (A11008, 1:200 dilution, Invitrogen), Alexa 488-conjugated anti-rat IgG (A11006, 1:200 dilution, Invitrogen), Alexa 488-conjugated anti-chick IgG (A11039, 1:200 dilution, Invitrogen), Cy5-conjugated anti-mouse IgG (A10524, 1:200 dilution, Invitrogen), Cy5-conjugated anti-rabbit IgG (A10523, 1:200 dilution, Invitrogen).<br>All the antibodies used for these studies are listed in the manuscript as well.
Validation	The specificity of each antibody was validated by the manufacturer, and provided on the website.<br>mouse anti-MF-20 antibody (MF-20, https://dshb.biology.uiowa.edu/MF-20), mouse anti-myoD (MA1-41017, https://www.thermofisher.com/antibody/product/MYOD-Antibody-clone-5-8A-Monoclonal/MA1-41017), rabbit anti-myogenin (ab124800, https://www.abcam.com/myogenin-antibody-epr4789-ab124800.html), rabbit anti-βIIIT (ab18207, https://www.abcam.com/beta-iii-tubulin-antibody-ab18207.html), rabbit anti-NF (N4142, https://www.sigmaaldrich.com/catalog/product/sigma/n4142?lang=en&region=US), rabbit anti-GFAP (ab7260, https://www.abcam.com/gfap-antibody-ab7260.html), rat anti-AChR antibody (ab24719, https://www.abcam.com/nicotinic-acetylcholine-receptor-alpha-1--3--5-antibody-210-ab24719.html), rabbit anti-vWF (A0082, https://www.agilent.com/cs/library/packageinsert/public/SSA0082IVD-US_01.pdf), mouse anti-α-SMA (sc-53015, https://datasheets.scbt.com/sc-53015.pdf), chicken anti-NF (ab4680, https://www.abcam.com/neurofilament-heavy-polypeptide-antibody-ab4680.html), rabbit anti-HLA (ab52922, https://www.nature.com/articles/s41598-018-29968-5), mouse anti-HNA (clone 3E1.3, MAB4383, http://www.emdmillipore.com/US/en/product/Anti-Nuclei-Antibody-clone-3E1.3,MM_NF-MAB4383?bd=1), Texas Red-conjugated anti-mouse IgG (TI-2000, https://vectorlabs.com/texas-red-horse-anti-mouse-igg-antibody.html), Texas Red-conjugated anti-rabbit IgG (TI-1000, https://vectorlabs.com/texas-red-goat-anti-rabbit-igg-antibody.html), Texas Red-conjugated anti-rat IgG (TI-9400, https://www.citeab.com/antibodies/3379433-ti-9400-texas-red-goat-anti-rat-igg-antibody), Alexa 488-conjugated anti-rabbit IgG (A11008, https://www.thermofisher.com/

antibody/product/Goat-anti-Rabbit-IgG-H-L-Cross-Adsorbed-Secondary-Antibody-Polyclonal/A-11008), Alexa 488-conjugated anti-rat IgG (A11006, https://www.thermofisher.com/antibody/product/Goat-anti-Rat-IgG-H-L-Cross-Adsorbed-Secondary-Antibody-Polyclonal/A-11006), Alexa 488-conjugated anti-chick IgG (A11039, https://www.thermofisher.com/antibody/product/Goat-anti-Chicken-IgY-H-L-Secondary-Antibody-Polyclonal/A-11039), Cy5-conjugated anti-mouse IgG (A10524, https://www.thermofisher.com/antibody/product/Goat-anti-Mouse-IgG-H-L-Cross-Adsorbed-Secondary-Antibody-Polyclonal/A10524), Cy5-conjugated anti-rabbit IgG (A10523, https://www.thermofisher.com/antibody/product/Goat-anti-Rabbit-IgG-H-L-Cross-Adsorbed-Secondary-Antibody-Polyclonal/A10523).

Eukaryotic cell lines

Policy information about cell lines

Cell line source(s)	ReNCell VM were obtained from Millipore. Human muscle progenitor cells were obtained from biopsies of human gracilis muscle (from 51- ad 62-year-old women, de-identified).
Authentication	Cell lines were not additionally authenticated.
Mycoplasma contamination	Cell lines were not additionally tested for mycoplasma.
Commonly misidentified lines (See ICLAC register)	No commonly misidentified cell lines were used.

Animals and other organisms

Policy information about studies involving animals; ARRIVE guidelines recommended for reporting animal research

Laboratory animals	RNU rats (male, 10-12 weeks old, Charles River Laboratory, Wilmington, MA) were used in this study.
Wild animals	This study did not involve wild animals.
Field-collected samples	No field-collected samples were used in the study.
Ethics oversight	All animal experiments were performed in accordance with a protocol approved by the Institutional Animal Care and Use Committee (IACUC) at Wake Forest University.

Note that full information on the approval of the study protocol must also be provided in the manuscript.
